# Research on the Expression of Immune-Related Genes at Different Stages in the Third-Instar Larvae of *Spodoptera frugiperda* Infected by *Metarhizium rileyi*

**DOI:** 10.3390/insects16020199

**Published:** 2025-02-12

**Authors:** Pengfei Xu, Zhan He, Xuyuan Gao, Xianru Zeng, Dewei Wei, Xiuzhen Long, Yonghao Yu

**Affiliations:** 1 Key Laboratory of Green Prevention and Control on Fruits and Vegetables in South China Ministry of Agriculture and Rural Affairs, Nanning 530007, China; xpfei0306@163.com (P.X.); happy-hezhan@163.com (Z.H.); gaoxuyuan1989@163.com (X.G.); zxr@gxaas.net (X.Z.); wdw@gxaas.net (D.W.); 2 Guangxi Key Laboratory of Biology for Crop Diseases and Insect Pests, Nanning 530007, China; 3 Plant Protection Research Institute, Guangxi Academy of Agricultural Sciences, Nanning 530007, China

**Keywords:** *Metarhizium rileyi*, *Spodoptera frugiperda*, infection process, innate immunity, RNA-seq

## Abstract

*Spodoptera frugiperda* is a major migratory agricultural pest that poses a significant threat to global crop safety. *Metarhizium rileyi* has emerged as an effective biocontrol agent against lepidopteran pests. In this study, we examined the immune responses of third-instar *S. frugiperda* larvae at various stages of an *M. rileyi* infection. Using RNA-seq and microscopic observation, we identified the immune-related pathways enriched at different infection stages, which were further validated by a qRT-PCR. Our findings revealed the following immune responses during infection: During the stage when *M. rileyi* penetrated the host cuticle (0–48 h), the genes related to energy metabolism, detoxification, and melanization were upregulated. Meanwhile, the TOLL and IMD signaling pathways were activated to counter the infection. During the stage of *M. rileyi*’s internal infection (48–96 h), which was the peak expression period of the immune-related genes, cellular immunity predominated. Hemocytes encapsulated and phagocytosed the hyphal bodies. Phagocytosis was enhanced through the upregulation of the genes related to ROS and the melanization-related genes, as well as the genes involved in insect hormone biosynthesis. During the stage when *M. rileyi* grew from the inside to the outside of the host (96–120 h), immune system paralysis resulted in host mortality. These findings deepen our understanding of the immune interactions between *M. rileyi* and *S. frugiperda*, support the potential of *M. rileyi* as an effective biocontrol agent, and provide a theoretical foundation for the development of targeted biopesticides for pests using biotechnological approaches.

## Simple Summary

*Spodoptera frugiperda* has caused severe economic losses in the agricultural sectors of various countries worldwide. Compared with chemical insecticides, *Metarhizium rileyi* represents an environmentally friendly approach to pest control, averting environmental pollution and negative impacts on human health. However, the application of *Metarhizium rileyi* in controlling *Spodoptera frugiperda* involves issues such as having a long action time and not having a highly efficient infectivity against all the developmental stages of the pest. We primarily investigated the variations in the immune responses of third-instar *Spodoptera frugiperda* larvae at different infection stages. The results demonstrated that at different infection stages, *Spodoptera frugiperda* expresses different genes in various metabolic pathways to counteract this fungal infection. The peak expression period for immune-related genes is 48–96 h. This study provides a theoretical foundation for constructing engineered strains with a stronger infectivity.

## 1. Introduction

*Metarhizium rileyi* (Farlow) Kepler, previously classified as *Nomuraea rileyi* (Farlow) Samson [1], is a widely distributed entomopathogenic fungus that infects over 60 species of Lepidoptera pests under natural conditions, showing a particularly high virulence against noctuid pests such as *Helicoverpa armigera*, *Spodoptera exigua*, *Spodoptera litura*, and *Spodoptera frugiperda* [2,3,4]. It plays a crucial role in biological pest control and has significant potential for practical applications. Upon encountering a host’s body surface, *M. rileyi* conidia secrete mucilaginous substances and adhesive proteins to facilitate attachment. Under favorable conditions, the conidia germinate, forming germ tubes that develop into appressoria or infection pegs [5,6], which secrete hydrolytic enzymes to degrade the host’s cuticle and penetrate the integument via both enzymatic action and mechanical pressure [7,8,9,10]. As a dimorphic pathogen, *M. rileyi* undergoes a morphological transition upon penetrating the host’s integument and entering the hemocoel. To overcome or evade the host’s immune response, the fungus shifts from hypha growth to the formation of hyphal bodies, which proliferate rapidly by budding or lysis, utilizing the host’s nutrients [11,12]. Once the density of the hyphal bodies reaches a critical threshold, the growth shifts from budding to apical expansion, resulting in a mycelial formation that disrupts the host tissue structure. After the host’s death, the mycelium emerges to form conidia, which can then attach to the integument of a new host, continuing the cycle of infection [13].

To defend against pathogenic microorganisms, insects activate their innate immune system, which consists of both cellular and humoral immunities that work in tandem to combat infections [14]. Pathogen-associated molecular patterns (PAMPs) in pathogens are recognized by insect pattern recognition receptors (PRRs), triggering the activation of the innate immune response [15]. The cellular immune response involves phagocytosis, nodulation, and encapsulation, which are processes mediated by hemocytes to eliminate invading pathogens [16]. In contrast, humoral immunity primarily targets foreign microbes through the synthesis of antimicrobial peptides (AMPs), the production of reactive oxygen intermediates (ROIs) or reactive nitrogen intermediates (RNIs), and the activation of the prophenoloxidase-activating system (proPO-AS), which regulates coagulation and melanization [17,18,19]. Among these defenses, AMPs are the principal effectors of insect humoral immunity. Their synthesis is rapidly induced upon the recognition and binding of PAMPs by PRRs, with production occurring in hemocytes, fat bodies, the epidermis, the midgut, and other tissues [20].

*Spodoptera frugiperda*, commonly known as the fall armyworm, belongs to the order Lepidoptera, family Noctuidae, and genus *Spodoptera*. It is a major migratory pest of global significance [21,22]. The current research on immune-related genes in *S. frugiperda* is relatively advanced, but studies identifying the pathways and genes that rapidly change and play a role at different stages of the infection process are not yet available. In this study, we conducted RNA sequencing on third-instar *S. frugiperda* larvae at various time points following infection with *M. rileyi* CDTLJ1. By comparing different experimental groups and combining RNA-seq data with microscopic observations of the infection process, we identified the genes with significant differential expression at different infection stages. A KEGG enrichment analysis was performed to uncover the primary pathways enriched with differentially expressed genes (DEGs), and qRT-PCR was used to validate these findings. Our results provide deeper insights into the interactions between insect hosts and pathogenic fungi, contributing to a better understanding of insect immune defense mechanisms against pathogenic fungi. Additionally, our findings could inform strategies for using the strain CDTLJ1 as a biocontrol agent to manage *S. frugiperda* populations and reduce crop damage.

## 2. Materials and Methods

### 2.1. Insect Cultivation

*S. frugiperda* individuals were collected from corn plants in fields in the Wuming District, Nanning City, Guangxi, and subsequently reared in the laboratory on corn leaves. After the insects emerged and laid eggs, a laboratory colony was established to provide test subjects for the experiments. For the indoor rearing conditions, a temperature of 27 ± 1 °C, a relative humidity ranging from 70% to 90%, and a photoperiod of 14 h of light and 10 h of darkness (14L:10D) were maintained.

### 2.2. Fungal Strain Cultivation

The *M. rileyi* CDTLJ1 used in this experiment is maintained at the Institute of Plant Protection at the Guangxi Academy of Agricultural Sciences, with the conidia suspended in 20% glycerol and stored at −80 °C. For culturing, the strain was inoculated onto an SMAY medium (maltose 40 g, peptone 10 g, yeast extract 10 g, agar 18 g, and distilled water 1000 mL) and incubated in an artificial climate chamber at 26 ± 1 °C, with a relative humidity of 80 ± 5%, and a 12L:12D photoperiod. The growth of the strain was relatively slow. In the early stage, it was mainly in the form of white mycelium. After 10 days of cultivation, green conidia formed and were stored at 4 °C for future use.

### 2.3. Infection via Immersion Method

The conidial powder of the test strain grown on the SMAY medium was collected using a sterilized soft-bristled brush and transferred into a sterilized 50 mL centrifuge tube. A 0.05% Tween-80 sterile solution was added, and the mixture was vortexed. The suspension was then filtered through four layers of sterilized lens paper to remove the mycelium and residual culture medium. The resulting filtrate was diluted with the 0.05% Tween-80 sterile solution to achieve a final concentration of 1 × 10^8^ conidia/mL. Uniform-sized third-instar *S. frugiperda* larvae were selected for immersion treatment. The larvae were immersed in the conidial suspension for 5 s, placed on sterilized filter paper to absorb excess moisture, and then individually transferred to sterilized Petri dishes (d = 7.5 cm) containing moist filter paper. Fresh corn leaves were provided as food, with leaves replaced daily. A 0.05% Tween-80 sterile water solution was used as the control. After treatment, the larvae were placed in an artificial climate chamber at 26 ± 1 °C, with a relative humidity of 80% ± 5%, and a 14L:10D photoperiod. The control and test samples were collected 24, 48, 72, 96, and 120 h post-inoculation, with 3 larvae per group and 3 replicates. The samples were immediately frozen in liquid nitrogen and stored at −80 °C for future analysis.

### 2.4. Preparation and Observation of Samples at Different Infection Stages of S. frugiperda by M. rileyi

Third-instar *S. frugiperda* larvae were infected via immersion (as described in Section 2.3). The selection of the observation time referred to the results of previous studies conducted by this research group [23]. The larvae 24 h post-infection were fixed in 2.5% glutaraldehyde for pre-fixation and left overnight at 4 °C. After fixation, the larvae were rinsed three times with a 0.1 M phosphate buffer for 15 min each. The samples were then subjected to a gradient ethanol dehydration series (30%, 40%, 50%, 60%, 70%, 80%, 90%, 95%, 100%, 100%, 100%), with each concentration applied for 15–20 min. The samples were dried in a critical-point dryer for 1 h, mounted on a stage, sputter-coated with gold, and examined using a scanning electron microscope (FEI Quattro S, ThermoElectron, Brno, The Czech Republic).

At 48, 72, and 96 h post-infection, the larvae were anesthetized with carbon dioxide. A hind leg was removed from each anesthetized larva using sterilized scissors, and 10 μL of hemolymph was collected and mixed with 10 μL of an anticoagulant (glucose 9.90 g, sodium citrate 4.41 g, citric acid 2.73 g, sodium chloride 1.81 g, EDTA 1.86 g, and ultrapure water 500 mL). The hemolymph was observed and photographed under a microscope at 40× magnification.

The larvae at 0 and 120 h post-infection were observed and photographed using a standard optical microscope. Three larvae per group were used, with three replicates.

### 2.5. RNA Extraction

The tissues stored at −80 °C for at least 24 h were taken out and ground into powder in a mortar pre-cooled in liquid nitrogen and then divided into RNase-free EP tubes. For one tissue sample, 1 mL of TRE Reagent was added (Shanghai Sangon Biotech Co., Ltd., Shanghai, China), and the sample was homogenized with a homogenizer, followed by the addition of 400 μL of RNase-free ddH_2_O, then mixed by inversion and left at room temperature for 5 min before undergoing centrifugation at 12,000 rpm for 15 min. The upper aqueous phase was transferred to another RNase-free EP tube, and an equal volume of isopropanol was added and mixed, then left at room temperature for 20 min. After centrifugation at 12,000 rpm for 10 min, the supernatant was discarded. The pellet was washed with 1 mL of 75% ethanol and centrifuged at 12,000 rpm for 3 min, the supernatant was discarded, and then this step was repeated. After air-drying at room temperature for 5 min, 50 µL of RNase-free ddH_2_O was added to fully dissolve the RNA.

The RNA yield and preparation purity were analyzed via the measurement of the ratios A260/A280 and A260/A230 with a Nanodrop 2000 spectrophotometer (Gene Company Limited, Shanghai, China). The RNA preparations were conserved at −80 °C.

### 2.6. RNAseq

The samples were sent to Novogene for transcriptome sequencing. RNA integrity and quantity were assessed using an Agilent 2100 bioanalyzer, and only high-quality RNA samples (RIN ≥ 4.0) were used for library construction. RNA purification, reverse transcription, library construction, and sequencing were conducted at Novogene (Beijing, China). Following library construction, preliminary quantification was performed with a Qubit 2.0 Fluorometer, adjusting the library concentration to 1.5 ng/μL. The insert size was then determined using an Agilent 2100 bioanalyzer (Novogene Co., Ltd., Beijing, China), and qRT-PCR was employed to accurately quantify the effective concentration of the library (effective concentration > 1.5 nM) to ensure library quality. After quality control, the 5′ end of the library was phosphorylated and circularized. The circularized library underwent rolling circle amplification to generate DNA nanoballs (DNBs), which were loaded onto a flow cell and sequenced using the DNBSEQ-T7 platform (MGI Technology Co., Ltd., Shenzhen, China).

### 2.7. Data Analysis

The raw reads underwent quality control to obtain clean reads, which were then assessed for Q20, Q30, and GC content. All subsequent analyses were based on high-quality clean data. HISAT2 v2.0.5 was used to align the paired-end clean reads with the reference genomes. Feature Counts (1.5.0-p3) was employed to calculate the FPKM for each gene, and a differential expression analysis was conducted using DESeq2 (1.20.0) with a Q-value ≤ 0.05. Genes with |log2FC| > 1 and Q-value ≤ 0.05 were considered significantly differentially expressed genes (DEGs). Additionally, a KEGG functional enrichment analysis was performed to identify the DEGs significantly enriched in metabolic pathways, using a Bonferroni-corrected *p*-value ≤ 0.05 compared to the whole transcriptome background. The KEGG pathway analyses were conducted using cluster Profiler (3.8.1).

### 2.8. Gene Expression Analysis

To investigate the expression of immune-related genes in third-instar *Spodoptera frugiperda* larvae at different stages of infection by *Metarhizium rileyi*, we compared the DEGs at different consecutive infection time points (|log2FC| > 1 and Q-value ≤ 0.05). A KEGG enrichment analysis was performed to identify the immune-related pathways, and representative genes were selected for further analysis.

### 2.9. RT-qPCR Validation

To quantify the expression levels of immune genes at different infection stages of *S. frugiperda* by *M. rileyi*, samples were collected at 24, 48, 72, 96, and 120 h post-infection and at 0, 24, 48, 72, 96, and 120 h post-Tween-80 treatment. The method was the same as that in Section 2.3. Total RNA was extracted using a TRE Reagent (see Materials and Methods, Section 2.4). For each sample, cDNAs were synthesized from 1 μg of RNA using the Trans Script Uni All-in-One First-Strand cDNA Synthesis Super Mix for qPCR (One-Step gDNA Removal), following the manufacturer’s protocol (TransGen Biotech, Beijing, China). A qPCR was performed with ChamQ Universal SYBR qPCR Master Mix (Vazyme) on a Mic real-time PCR system (BMS) under the following conditions: 95 °C for 30 s, followed by 40 cycles at 95 °C for 15 s and 60 °C for 30 s, with a melting curve from 65 to 95 °C. Each sample was analyzed with three biological replicates and three technical replicates. Primers were designed using Primer Premier 5 (version 5. 0), and the primers used for the qPCR are listed in Table S1. RPS24 was used as the internal control, and quantification was performed using the ΔΔCt method. All the statistical analyses were performed using IBM SPSS software (version 22. 0).

## 3. Results

### 3.1. Data Quality Control and Alignment with the Reference Genome

The control group and samples collected at 24, 48, 72, 96, and 120 h post-infection were designated as CK3, A3, B3, C3, D3, and E3, respectively. To ensure data accuracy, we performed quality control on the sequencing data and aligned it with the reference genome for *S. frugiperda* available in the NCBI database. After the removal of low-quality reads, the number of clean reads for each sample exceeded 42,511,856, with a data retention rate of over 95%. The mapping rate to the reference genome ranged from 64 to 72% for the 120 h treatment group and was above 77% for all the other groups. The mapping rate decreased with the longer treatment times, suggesting an increasing presence of hyphal bodies in the hemocoel of *S. frugiperda* as the treatment progressed. The 120 h treatment group had a significantly lower mapping rate compared to the other groups (*p* < 0.01), indicating that the highest number of hyphal bodies could be found at this time point, which is consistent with our previous findings [21]. The error rate for each sample was 2%, with the Q20 data exceeding 98.76%, Q30 data exceeding 96.80%, and GC content exceeding 44.13% (as shown in Table 1), confirming that the data quality met the requirements for the subsequent analyses.

### 3.2. Sample Correlation Analysis

**Figure 1:**
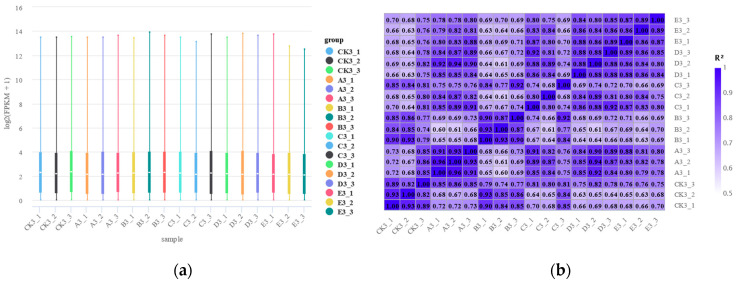
(**a**) Boxplot of gene expression distribution. (**b**) Heatmap of sample correlations.

Due to the influence of sequencing depth and gene length, gene expression values in RNA-seqs are typically normalized using FPKM to account for both these factors, enabling the quantitative analysis of all the genes in each sample. The distribution of gene expression levels across the samples is shown in Figure 1a, with the number and distribution of gene expressions consistent among all groups. The correlation analysis between the samples is shown in the heatmap in Figure 1b. Except for sample C3-3, the square of the Pearson correlation coefficient (R^2^) between all groups exceeds 0.8, indicating good repeatability within the groups. The R^2^ values between all the samples in groups D and E are greater than 0.8, suggesting that the gene expression patterns in *S. frugiperda* larvae are quite similar 96 and 120 h post-infection with *M. rileyi*.

### 3.3. Observation of the Microscopic Infection Process of S. frugiperda by M. rileyi

**Figure 2:**
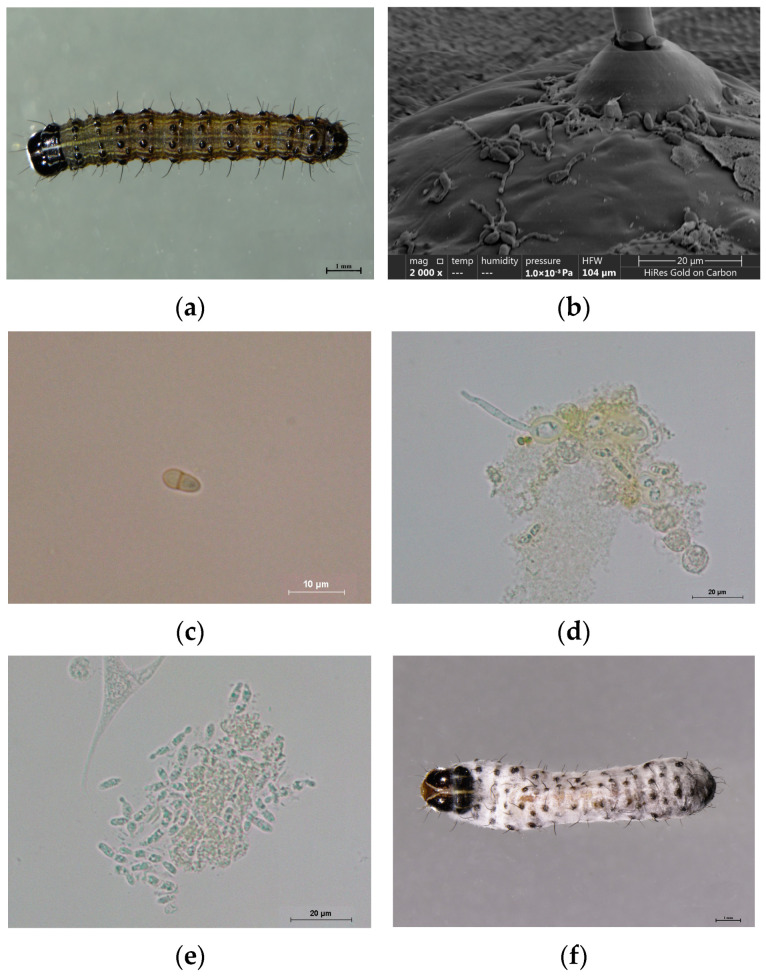
Microscopic observation of the infection process of strain CDTLJ1 in *S. frugiperda* third-instar larvae at (**a**) 0 h post-infection (2×), (**b**) 24 h post-infection (2000×), (**c**) 48 h post-infection (600×), (**d**) 72 h post-infection (600×), (**e**) 96 h post-infection (600×), and (**f**) 120 h post-infection (2×). Note: the figure in the brackets indicates the magnification of the microscope.

To analyze the infection patterns of the strain CDTLJ1 on third-instar *S. frugiperda* larvae at different stages, we performed microscopic observations of the entire infection process using optical and scanning electron microscopy. The results are shown in Figure 2.

Before infection, no hyphae were observed on the surface of the *S. frugiperda* larvae (Figure 2a). At 24 h post-infection, the strain CDTLJ1 had attached to the larvae’s surface in large numbers, with the hyphae elongating and penetrating the host’s cuticle at their tips (Figure 2b). By 48 h post-infection, the strain CDTLJ1 had penetrated the host’s cuticle, and hyphal bodies were first observed in the hemolymph as short, rod-shaped, segmented structures, though they were present in small numbers (Figure 2c). At 72 h post-infection, the larvae used a large number of hemocytes to phagocytose or encapsulate the hyphal bodies, while the hyphal bodies evaded the larval immune response by extending germ tubes from within phagosomes or capsules (Figure 2d). At 96 h post-infection, the hyphal bodies reached their peak division rate, aggregating in large numbers. The majority of hemocytes were degraded, and the insect’s hemolymph became turbid (Figure 2e). Finally, at 120 h post-infection, the insect died, and hyphae emerged from within the body, covering the entire surface of the insect (Figure 2f).

### 3.4. Immune Expression Patterns of S. frugiperda at Different Stages of M. rileyi Infection

**Figure 3:**
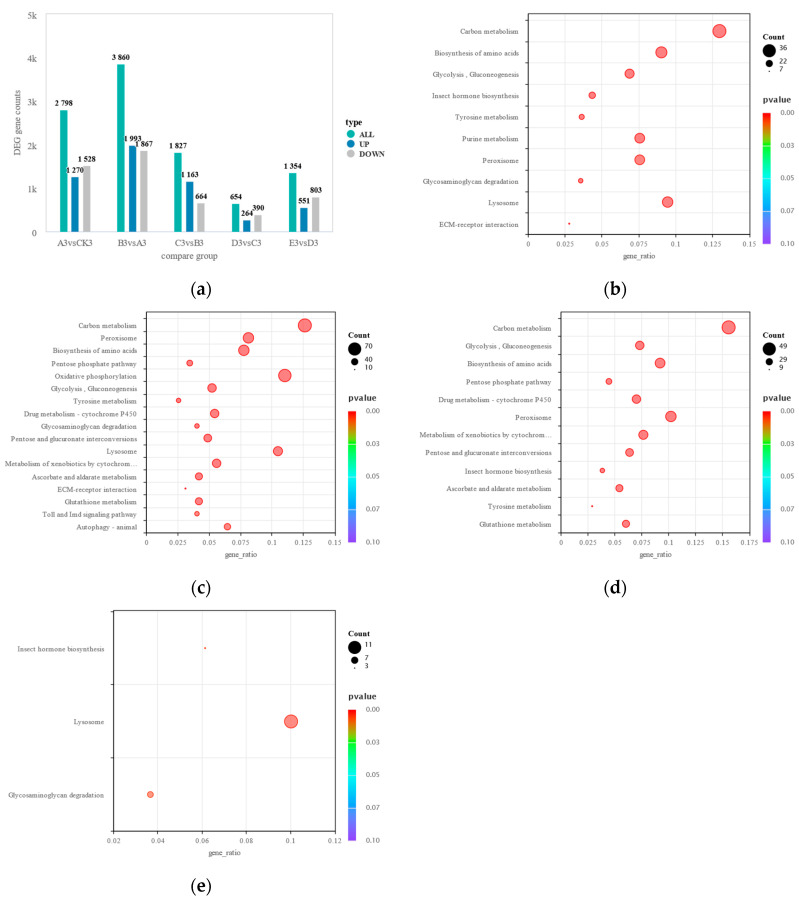
(**a**) Bar chart showing the number of differential genes in different comparison groups. KEGG pathway enrichment analysis at different stages post-infection: (**b**) 0–24 h post-infection, (**c**) 24–48 h post-infection, (**d**) 48–72 h post-infection, and (**e**) 72–96 h post-infection.

To analyze the expression of the immune-related genes in *S. frugiperda* at different stages of infection by *M. rileyi* CDTLJ1, we performed a differential gene expression analysis across various comparison groups. The results showed that the numbers of DEGs at the five infection stages were 2798, 3860, 1872, 654, and 1354, respectively. During the first three stages of infection (0–72 h), the number of upregulated genes exceeded 1163 at each stage, significantly higher than the 264 genes at 72–96 h and the 551 genes at 96–120 h. This suggests that, compared to the earlier stages, the biological activity of *S. frugiperda* was more stable during the 72–120 h period (Table 2 and Figure 3a), consistent with the findings in Section 3.2, where gene expression patterns were similar at 96 and 120 h post-infection. Notably, the 24–48 h stage exhibited the highest number of both upregulated and downregulated genes, indicating that the strongest stress response in the *S. frugiperda* larvae occurred during this period following an infection by the strain CDTLJ1.

A KEGG pathway enrichment analysis was conducted on the selected DEGs, focusing on the immune-related pathways. The results revealed that the number of immune-related pathways enriched at each of the five stages of infection was 10, 17, 12, 4, and 0, respectively, with the total number of enriched genes being 184, 506, 270, 28, and 0. This indicates that over the first 72 h post-infection, the immune response in the *S. frugiperda* larvae fluctuated rapidly, with the most significant changes occurring during the 24–48 h stage. However, the immune response during the 72–96 h stage showed minimal changes and gradually stabilized (Table 3).

During the 0–24 h stage, the immune-related pathways that were enriched included carbon metabolism, the biosynthesis of amino acids, glycolysis/gluconeogenesis, insect hormone biosynthesis, tyrosine metabolism, purine metabolism, peroxisome, glycosaminoglycan degradation, the lysosome pathway, and the ECM–receptor interaction pathway. Among these, carbon metabolism had the highest number of DEGs (36) and the ECM–receptor interaction pathway had the fewest (7) (Figure 3b). Carbon metabolism is crucial in insect immunity, as the immune system requires energy for various functions, such as immune cell migration, signal transduction, and immune molecule synthesis [24]. In insect immunity, numerous proteins are involved in pathogen recognition, the activation of signaling pathways, and the stimulation of phagocytosis or humoral responses, and many peptides have direct antimicrobial activity. By 2014, over 150 insect AMPs had been isolated or identified, all of which were linked to amino acid biosynthesis [25,26,27]. Glycolysis/gluconeogenesis provides ATP under anaerobic conditions, serving as a key energy source for immune responses and providing precursors for the synthesis of proteins, lipids, nucleic acids, and polysaccharides [28]. The insect hormone biosynthesis pathway includes the production of 20-hydroxyecdysone (20E) and the juvenile hormone (JH); the JH acts as an immune activator, and 20E inhibits innate immunity through its receptor complex EcR-US [29]. The tyrosine metabolism pathway is involved in pigmentation and innate immune responses, with tyrosine hydroxylase (TH) converting tyrosine to dopamine, a precursor in melanin biosynthesis [30]. Studies suggest that purine metabolism, triggered by ATP, ADP, and other nucleotides, plays a role in immune signaling by activating pro-inflammatory responses through P2 purinergic receptors [31]. Peroxisomes, important in lipid metabolism and reactive ion regulation, also play a key role in immunity, with polyunsaturated fatty acids (PUFAs) serving as precursors for immune mediators [32]. The glycosaminoglycan degradation pathway aids in pathogen binding and antimicrobial peptide regulation [33], while lysosomes contribute to autophagy and degradation in engulfed pathogens [34]. Finally, the ECM–receptor interaction pathway regulates granulocytes and plasma cells, leading them to rapidly transition from a circulating non-adherent state to an interactive state upon the invasion of foreign pathogens and to adhere to the surface of these pathogens to combat the invasion [35].

During the 24–48 h stage, several immune-related pathways were enriched, including glycosaminoglycan degradation, the lysosome pathway, the ECM–receptor interaction pathway, autophagy (animal), Toll and IMD signaling, carbon metabolism, oxidative phosphorylation, glycolysis/gluconeogenesis, the peroxisome pathway, drug metabolism (cytochrome P450), pentose and glucuronate interconversions, tyrosine metabolism, the pentose phosphate pathway, the biosynthesis of amino acids, ascorbate and aldarate metabolism, the metabolism of xenobiotics by cytochrome P450, and glutathione metabolism. Among these, carbon metabolism had the highest number of DEGs (70), while the ECM–receptor interaction pathway had the fewest (10) (Figure 3c). Some pathways enriched at this stage were the same as those observed during the 0–24 h stage, indicating that during the 24–48 h stage, there were significant changes in gene expression within these pathways compared to the previous stage, and the immune response pattern of the *S. frugiperda* larvae also underwent corresponding alterations. Additionally, several new immune-related pathways emerged at this stage.

The pattern recognition receptor PGRP-LE, which recognizes diaminopimelic acid-type peptidoglycan, induces autophagy and prevents pathogen growth within cells, thereby enhancing the host’s immune defense [36]. Toll-like receptors in the Toll and IMD signaling pathways detect infections and activate inflammatory and antimicrobial innate immune responses. In the IMD pathway, Gram-negative bacterial infections trigger the expression of AMPs [37,38]. The oxidative phosphorylation pathway generates reactive oxygen species (ROS), which enhance the insect’s antioxidant capacity and protect against oxidative stress [39]. The pathways for cytochrome P450-mediated drug metabolism and the metabolism of xenobiotics are crucial in the detoxification of exogenous substances [40,41]. D-glucuronic acid in pentose and glucuronate interconversions plays a key role in the insect detoxification processes [42,43]. The pentose phosphate pathway produces NADPH, maintaining cellular redox balance, supporting various antioxidant defenses, and protecting the host from immune response-induced damage [44]. The ascorbate and aldarate metabolism pathways eliminate ROS and other harmful substances, with the inhibition of ascorbic acid significantly reducing infection resistance in larvae [45]. Lastly, the glutathione metabolism pathway regulates melanization in insect hemolymph, modulates humoral immune responses, and plays a significant role in insect detoxification and resistance to biological stress [46,47].

During the 48–72 h stage, several immune-related pathways were enriched, including carbon metabolism, glycolysis/gluconeogenesis, the pentose phosphate pathway, insect hormone biosynthesis, the peroxisome pathway, pentose and glucuronate interconversions, glutathione metabolism, tyrosine metabolism, ascorbate and aldarate metabolism, the biosynthesis of amino acids, drug metabolism (cytochrome P450), and the metabolism of xenobiotics by cytochrome P450. Among these, carbon metabolism had the highest number of DEGs (49), while tyrosine metabolism had the fewest (9) (Figure 3d).

During the 72–96 h stage, the key immune-related pathways enriched included insect hormone biosynthesis, lysosome function, and glycosaminoglycan degradation. The lysosome pathway exhibited the highest number of DEGs (11), while glycosaminoglycan degradation had the fewest (3) (Figure 3e). No immune metabolism-related pathways were enriched during the 96–120 h stage. At the 72–120 h stage, the expression of 18 immune-related genes across three enriched metabolic pathways exhibited significant differences. During this period, the immune expression pattern of the insect stabilized.

### 3.5. Validation of DEGs by qRT-PCR

**Figure 4:**
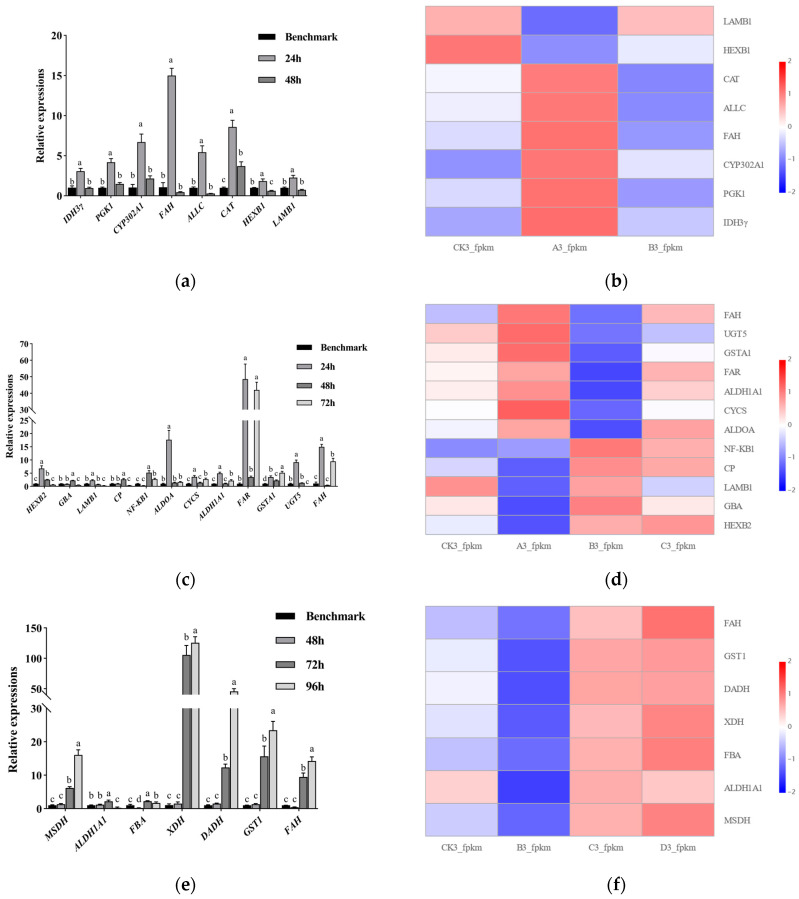

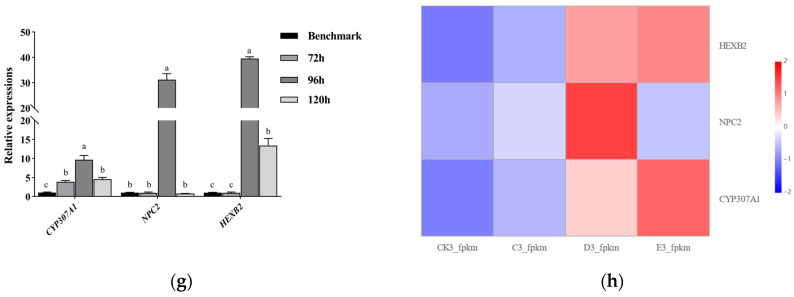
qRT-PCR expression validation and expression profiling of immune-related DEGs: (**a**,**b**) 0–24 h post-infection, (**c**,**d**) 24–48 h post-infection, (**e**,**f**) 48–72 h post-infection, and (**g**,**h**) 72–96 h post-infection. Note: Benchmark refers to the relative expression levels of the control groups at 24, 48, 72, 96, and 120 h after normalization, respectively. Different lowercase letters indicate significant differences (*p* < 0.05).

We validated the expression of DEGs using qRT-PCR, selecting one candidate gene from each immune-related pathway at each of the five infection stages, resulting in a total of 25 candidate genes for validation (Table S2). These genes were further analyzed to assess their potential roles in the cellular immune defense system of *S frugiperda* when infected by *M rileyi* CDTLJ1. To investigate the gene expression changes at each infection stage, we selected samples before and after each time point, as well as non-inoculated control samples. The qPCR validation results are presented in Figure 4.

#### 3.5.1. In the 0–24 h Infection Stage

The qRT-PCR results (Figure 4a,b) showed a significant upregulation of *IDH3γ*, *PGK1*, *CYP302A1*, *FAH*, *ALLC*, *CAT*, *HEXB1*, and *LAMB1* at 24 h compared to the Benchmark, with *FAH* and *CAT* exhibiting the highest expression levels, upregulated by 14.99 and 8.58 times, respectively. *FAH*, a terminal rate-limiting enzyme in the tyrosine catabolic pathway, plays a key role in the tanning and melanization of an insect cuticle [48]. *CAT*, a critical enzyme in the peroxisome pathway, functions as a potent antioxidant by breaking down ROS [49]. While the expression patterns of genes from various immune-related pathways generally aligned with the transcriptome data, slight discrepancies were noted, particularly for *HEXB1* and *LAMB1*, which were upregulated at 24 h, contrary to the transcriptome findings.

#### 3.5.2. In the 24–48 h Infection Stage

The qRT-PCR results (Figure 4c,d) revealed a significant downregulation of *HEXB2*, *LAMB1*, *ALDOA*, *CYCS*, *ALDH1A1*, *FAR*, *GSTA1*, *UGT5*, and *FAH* at 48 h compared to the 24 h time point. In contrast, *NF-KB1*, *GBA*, and *CP* were upregulated. Notably, *FAR* exhibited an expression level at 48 h that was 3.51 times higher than the Benchmark, with its expression at 24 and 72 h showing an even greater upregulation (48.53 and 42.01 times that of Benchmark, respectively). *NF-KB1* is a crucial gene in the Toll and IMD signaling pathways, responsible for activating innate immune responses and regulating antimicrobial peptide expression. In *Drosophila melanogaster*, the activation of the IMD and Toll pathways is initiated by pattern recognition receptors (PRRs) recognizing microbial pathogen-associated molecular patterns (PAMPs), which leads to the nuclear translocation of *NF-KB* transcription factors (e.g., Dorsal and Relish), subsequently inducing an innate immune response [50,51]. *GBA* and *CP* are involved in the lysosome and autophagy (animal) pathways, respectively. Autophagy, distinct from the Toll, IMD, and JAK-STAT pathways [36,52], facilitates the degradation of damaged organelles, engulfed materials, and protein aggregates through lysosomal fusion, contributing to cellular homeostasis [53,54]. The upregulation of *GBA* and *CP* at 48 h suggests the activation of cellular autophagy in this stage. *FAR*, enriched in the peroxisome pathway, plays a role in long-chain primary fatty alcohol biosynthesis, catalyzing the transformation of fatty acids into fatty alcohols using NADPH. These fatty alcohols, as precursors to wax esters, contribute to an insect cuticle, enhancing resistance to pathogenic microorganisms [55,56,57].

The expression patterns of *HEXB2* and *LAMB1* deviated slightly from the transcriptome data, showing a downward trend at 48 h, despite their roles in the 0–24 h stage. *HEXB2*, involved in the Glycosaminoglycan degradation pathway, inhibits the growth of pathogenic fungi. In silkworms infected with *Exorista bombycis* and *Nosema bombycis*, *HEXB* expression was enhanced in granulocytes, supporting its role in host defense. *HEXB* is upregulated in the early stages of infection and downregulated in the later stages, which is consistent with our qRT-PCR results [58,59]. *LAMB1*, a glycoprotein critical in basement membrane formation, cell adhesion, and receptor binding, shows a similar expression pattern to *HEXB2*. In *Crassostrea gigas*, β-integrins can bind to laminin proteins, playing a key role in immune responses such as phagocytosis, migration, and encapsulation [60,61]. At the 24–48 h stage, among the 12 immune-related genes analyzed, 9 were downregulated. Interestingly, five of these genes (*CYCS*, *ALLC*, *FAR*, *GSTA1*, and *FAH*) showed significant upregulation at both the 24 and 72 h time points, despite being downregulated at 48 h.

#### 3.5.3. In the 48–72 h Infection Stage

The qRT-PCR results (Figure 4e,f) indicated a significant upregulation of *MSDH*, *ALDH1A1*, *ALDOA*, *XDH*, *DADH*, *GST1*, and *FAH* at 72 h compared to 48 h. Notably, the expression levels of *MSDH*, *XDH*, *DADH*, *GST1*, and *FAH* were even higher at 96 h than at 72 h. Relative to the control (Benchmark) group, these genes were upregulated by more than 6.17 times, with *XDH* showing particularly high upregulation of 105.52 and 125.71 times that of the Benchmark at 72 and 96 h, respectively. These findings were consistent with the sequencing data.

*MSDH* is a key gene in the carbon metabolism pathway and encodes a mitochondrial enzyme that catalyzes the NAD-dependent oxidation of methylmalonate semialdehyde (MMSA) and malonate semialdehyde (MSA) into propionyl-CoA and acetyl-CoA, respectively, in a two-step process. Acetyl-CoA is subsequently utilized in the tricarboxylic acid (TCA) cycle to generate ATP, providing energy for immune processes [62,63,64]. *XDH* is involved in both the peroxisome and purine metabolism pathways, catalyzing the conversion of xanthine to uric acid, which helps eliminate ROS and peroxynitrite during innate immune responses. In *Arabidopsis*, *XDH* functions as a superoxide dismutase in infected tissues to combat pathogens, while, in healthy tissues, it acts as xanthine dehydrogenase, producing uric acid to neutralize ROS and mitigate oxidative stress [65,66]. DADH participates in the pentose and glucuronate interconversion pathway, metabolizing D-arabinitol and catalyzing NAD+-dependent oxidation to yield D-ribulose, a precursor to NADPH synthesis. NADPH is crucial in maintaining cellular glutathione in its reduced form, protecting cells from oxidative damage [67,68]. *GST1* is involved in multiple metabolic pathways, including glutathione metabolism, drug metabolism (via cytochrome P450), and the metabolism of xenobiotics, primarily functioning in the detoxification processes of an insect’s metabolism. In the armyworm, *GST1* is upregulated at both the transcriptional and protein levels in the midgut, where it degrades ingested toxic compounds, a function also observed in *Nilaparvata lugens* [69,70]. The *FAH* gene, identified at the 48–72 h stage, was also observed at the 0–24 h and 24–48 h stages, where it plays a role in melanization processes [48].

#### 3.5.4. In the 72–96 h Infection Stage

The qRT-PCR results (Figure 4g,h) revealed a significant upregulation of *CYP307A1*, *NPC2*, and *HEXB2* at 96 h compared to 72 h, with a fold change greater than 9.66 relative to the Benchmark. These findings were consistent with the sequencing data. *CYP307A1* is a key gene involved in insect hormone biosynthesis, specifically in the synthesis of ecdysone in *Drosophila* and *S*. *litura*. Ecdysone is one of the primary hormones that is critical to development and plays a role in promoting the innate immune responses of an insect [71,72,73]. *NPC2* is involved in the lysosome metabolic pathway, functioning as an intracellular cholesterol transporter. It plays a crucial role in regulating sterol homeostasis and in the synthesis of molting hormones [74]. The function of *HEXB2*, previously discussed in the 24–48 h stage, is also relevant here. Overall, the expression trends observed through the qRT-PCR were consistent with the RNA-seq data, underscoring the reliability of the sequencing results for use in subsequent functional studies.

#### 3.5.5. In the 96–120 h Infection Stage

Although many genes remained differentially expressed compared to the 96 h stage, the insect’s immune system appeared to be fully suppressed by 120 h, likely due to the insect’s death, and no immune-related genes were enriched.

## 4. Discussion

*Spodoptera frugiperda*, a widely distributed invasive pest, is primarily controlled using chemical pesticides, which raises concerns about environmental pollution, pesticide residues, and resistance development. These issues contribute to significant global economic losses, driving the search for alternative control strategies [3,21,75]. *Metarhizium rileyi*, a promising biological control agent, infects *S. frugiperda* and other noctuid pests without the risk of resistance development. However, limitations such as its slow pathogenicity and a lack of strains that are effective across all pest life stages hinder its widespread application [76,77]. Therefore, understanding the molecular interactions between *M. rileyi* and its insect hosts is crucial to improving the efficacy of entomopathogenic fungi. In this study, RNA-seq was used to analyze changes in host immune-related gene expression at different stages of infection by the strain CDTLJ1 in *S. frugiperda*. The findings shed light on the interactions between *M. rileyi* and *S. frugiperda* larvae, offering valuable insights that can be used in enhancing biocontrol strategies against this pest.

Like other organisms, insects present a complex immune response to fungal infections. However, unlike mammals, insects lack adaptive immunity and rely predominantly on their innate immune system, which includes physical barriers, humoral immunity, and cellular immunity, with humoral and cellular responses often working in tandem [78,79]. The immune response in *S. frugiperda* larvae varies according to the infection stage of the strain CDTLJ1, with distinct immune reactions occurring at different time points.

During the 0–24 h stage, the *M. rileyi* strain CDTLJ1 adheres to the cuticle of *S. frugiperda* larvae, germinates, forms germ tubes, and penetrates the host’s integument, breaching its physical immune barrier. The upregulation of immune-related pathways at this stage suggests an active host defense response against fungal invasion. Key genes such as *IDH3γ* and *PGK1* provide energy for the immune response. *IDH3γ* is a mitochondrial enzyme involved in the citric acid cycle [80], while *PGK1* plays a central role in glycolysis by catalyzing the conversion of 1,3-bisphosphoglycerate to 3-phosphoglycerate. In Drosophila, the knockdown of *PGK* leads to reduced ATP levels and the accumulation of ROS in the central nervous system of third-instar larvae [81]. These genes supply the ATP and metabolic precursors necessary for immune responses. The detoxification-related genes, such as *CYP302A1*, *ALLC*, and *CAT*, are also upregulated. *S. frugiperda* larvae utilize *CYP302A1* for detoxification, allowing them to adapt to host plants [82]. *ALLC* catalyzes the hydrolysis of allantoin to allantoic acid, a potent antioxidant that reduces oxidative DNA damage [83]. *CAT*, a strong antioxidant enzyme, degrades toxic ROS [49], indicating a robust response to ROS and other toxic metabolites generated during fungal penetration. *FAH* is involved in melanization and acts as a rate-limiting enzyme in the tyrosine metabolism pathway, generating large amounts of melanin during this stage. Studies have shown that melanin appears on the cuticle surface where the pathogen breaches the host’s exoskeleton during a fungal invasion [84], consistent with our findings. The extreme upregulation of *FAH* and the substantial synthesis of melanin suggest the extensive hyphal penetration of the host’s cuticle, in agreement with our earlier research, which identified the 16–24 h period as the peak for strain CDTLJ1 penetration [23].

By the 24–48 h stage, the strain CDTLJ1 has penetrated the host’s cuticle and differentiated into hyphal bodies within the hemolymph. At this stage, the majority of immune-related genes that are enriched are downregulated, with only *NF-KB1*, *GBA*, and *CP* showing upregulation. NF-KB1 is involved in both the Toll and IMD signaling pathways, which activate the *NF-KB* transcription factor and transport it to the nucleus, where it induces the expression of AMPs and other effector molecules [85]. *GBA* and *CP* are associated with the lysosome and autophagy (animal) metabolic pathways, respectively. The lysosome, in coordination with autophagosomes, degrades pathogens and apoptotic cells, playing a critical role in innate cellular immunity [86]. In addition to the upregulation of these three pathways, the other pathways related to energy metabolism, detoxification, encapsulation, and melanization were downregulated, with five genes showing downregulation only at 48 h. In the studies by Liu S et al., the injected *Metarhizium anisopliae* mycelium was not recognized by the immune system of *S*. *litura* larvae as an invader. Similarly, Yang X et al. found that the mycelium of *M*. *anisopliae* injected into *S. frugiperda* larvae was not detected by the host’s immune system as a pathogen [76,87]. In our previous research, we found that during this stage, the infection mode of the strain CDTLJ1 shifted from an in vitro to an in vivo infection, with conidia transforming into hyphal bodies, consistent with the above studies. This allows the pathogen to evade the host’s immune response, leading to the downregulation of many immune-related genes during this stage. Moreover, at 48 h post-infection, due to molting in the *S. frugiperda* larvae, the fungal hyphae were no longer visible under scanning electron microscopy, indicating that the penetration of the cuticle had ceased [23]. Consequently, the melanization-related gene *FAH* was significantly downregulated, and *FAR*, a gene involved in epidermal defense against pathogens, was also significantly downregulated during this stage.

At the 48–72 h stage, the *S. frugiperda* larvae utilize large numbers of hemocytes to engulf or encapsulate the hyphal bodies, forming a capsule. To evade the host’s hemocyte-mediated immunity, the hyphal bodies extend germ tubes from the phagosome or capsule. The immune-related genes identified are all upregulated at this stage, apart from *ALDH1A1* and *FBA*, which show relatively modest increases. The other five genes (*MSDH*, *XDH*, *DADH*, *GST1*, and *FAH*) exhibit an upregulation of more than 6.17 times compared to the control group, with the highest upregulation being 125.71 times that of the Benchmark. Furthermore, their expression at 96 h is higher than at 72 h. No immune-related genes were enriched during the 96–120 h stage, which indicates that the peak expression of immune-related genes in *S. frugiperda* larvae occurs between 48 and 96 h, during which time the immune interaction between the strain CDTLJ1 and the larvae is most intense. Observations of the hyphal bodies at this stage show that cellular immunity plays a major role, accompanied by the phagocytosis, aggregation, and the encapsulation of pathogens. Hemocytes form layers of membranes around the pathogens, and the encapsulated microbes are killed by the ROS and reactive nitrogen species (RNS) generated by the insect’s immune system, often resulting in suffocation or death, with melanization occurring [88,89,90]. In this study, *MSDH*, a key gene in the carbon metabolism pathway, provides energy for the immune response, while *XDH*, *DADH,* and *GST1* participate in antioxidant and detoxification pathways, neutralizing ROS and the other toxic secondary metabolites released by fungal infections and combating invading pathogens. *FAH*, involved in melanization, was also significantly upregulated, which aligns with the microscopic observations and immune responses of the larvae during this stage.

At the 72–96 h stage, large numbers of hemocytes adhere to, and aggregate on, the surface of the hyphal bodies, forming melanized nodules as an immune response against the pathogen [91]. At 96 h post-infection, *CYP307A1*, *NPC2*, and *HEXB* are significantly upregulated compared to 72 h, indicating a shift in immune strategy. Both *CYP307A1* and *NPC2* are involved in insect hormone synthesis, with *NPC2* expression increasing 31.2-fold at 96 h before returning to normal levels, highlighting the importance of insect hormones in the immune response during this stage. Studies by Marcela B. Figueiredo et al. on *Rhodnius prolixus* have shown that the addition of ecdysteroids enhances phagocytic activity, and Jennifer C. Regan et al. found that individuals lacking ecdysteroid-activated hemocytes showed defects in bacterial phagocytosis, underscoring the critical role of ecdysteroid regulation in cellular immunity [92,93]. Consistent with these findings, in this study, the *S. frugiperda* larvae showed a significant upregulation of the insect hormone synthesis genes during this stage, enhancing the phagocytic response.

At the 96–120 h stage, the insect dies as hyphae grow out from its cuticle and cover the entire insect. During this stage, the insect immune system is paralyzed, the hemolymph is filled with hyphal bodies, and no immune-related genes are enriched. In conclusion, these findings deepen our understanding of the interactions between *M. rileyi* and the host’s immune response, providing insights that could inform the development of novel strategies for the biological control of key agricultural pests, including *S. frugiperda*. By unraveling the complex network of interactions between insects and insect pathogenic fungi, we gained a better understanding of the mechanisms underlying insect immune defense.

## 5. Conclusions

At different stages of *M. rileyi* infection, the larvae of *S. frugiperda* adopt different immune strategies to combat the infection, and the related metabolic pathways and genes change rapidly. At five different infection stages, we selected a total of 25 related genes to verify the results of the RNA-seq analysis. The results showed the following: at the 0–24 h stage, the genes related to energy metabolism, detoxification, and melanization were upregulated; at the 24–48 h stage, as the strain CDTLJ1 evaded the host’s immune recognition, the expression levels of a large number of immune-related genes decreased, but the TOLL and IMD signaling pathways were activated; at the 48–72 h stage, cellular immunity played a major role, and the genes related to hemocyte phagocytosis and melanization were significantly upregulated; at the 72–96 h stage, the genes related to the elimination of ROS, melanization, and insect hormone biosynthesis were all significantly upregulated, enhancing the phagocytic effect of hemocytes; and at the 96–120 h stage, the larvae died, and the immune system was completely paralyzed. Our results contribute to a deeper understanding of the molecular mechanism behind *M. rileyi* infecting *S. frugiperda* and provide a theoretical basis for constructing engineered strains with stronger infectivity.

## Figures and Tables

**Table 1:** Summary of sample sequencing data quality.

Sample	Raw Reads	Clean Reads	Total Mapped	Error Rate	Q20	Q30	GC pct
CK3-1	43,367,022	42,511,856 (98.03%)	35,006,950 (82.35%)	2%	99.42%	97.92%	44.13%
CK3-2	50,717,152	49,925,924 (98.44%)	42,204,798 (84.53%)	2%	99.37%	97.75%	45.10%
CK3-3	62,432,376	60,696,498 (97.22%)	49,893,636 (82.2%)	2%	99.48%	98.12%	44.83%
A3-1	48,843,600	47,322,616 (96.89%)	40,117,946 (84.78%)	2%	99.51%	98.19%	46.07%
A3-2	61,079,356	59,185,064 (96.90%)	49,189,064 (83.11%)	2%	99.52%	98.24%	45.73%
A3-3	47,011,814	45,753,728 (97.32%)	38,312,114 (83.74%)	2%	99.46%	98.06%	45.63%
B3-1	60,211,080	58,783,004 (97.63%)	49,540,798 (84.28%)	2%	99.46%	98.07%	45.41%
B3-2	50,325,142	48,221,068 (95.82%)	39,938,058 (82.82%)	2%	99.55%	98.42%	45.77%
B3-3	55,220,764	53,710,008 (97.26%)	45,028,004 (83.84%)	2%	99.53%	98.35%	46.50%
C3-1	58,768,998	57,560,766 (97.94%)	47,322,624 (82.21%)	2%	99.50%	98.19%	45.30%
C3-2	47,417,674	46,437,988 (97.93%)	39,223,073 (84.46%)	2%	99.51%	98.18%	46.03%
C3-3	46,405,770	45,126,816 (97.24%)	38,276,680 (84.82%)	2%	98.76%	96.80%	48.89%
D3-1	47,435,408	46,579,502 (98.20%)	35,932,567 (77.14%)	2%	99.53%	98.26%	44.91%
D3-2	58,311,500	57,206,792 (98.11%)	47,609,851 (83.22%)	2%	99.51%	98.19%	45.85%
D3-3	56,390,606	54,498,098 (96.64%)	42,971,423 (78.85%)	2%	99.49%	98.16%	46.27%
E3-1	53,196,460	51,852,934 (97.47%)	34,356,599 (66.26%)	2%	99.49%	98.12%	47.24%
E3-2	46,973,698	45,790,100 (97.48%)	33,154,751 (72.41%)	2%	99.50%	98.16%	46.99%
E3-3	46,136,728	44,539,592 (96.54%)	28,797,986 (64.66%)	2%	99.50%	98.15%	47.02%

**Table 2:** Statistics for differential gene counts in different comparison groups.

Comparison	Up	Down	All
A3vsCK3	1270	1528	2798
B3vsA3	1993	1867	3860
C3vsB3	1163	664	1827
D3vsC3	264	390	654
E3vsD3	551	803	1354

**Table 3:** Statistics for immune-related pathways in different comparison groups.

Comparison	Immune-Related Pathways	SUM of Genes
A3vsCK3	10	184
B3vsA3	17	506
C3vsB3	12	270
D3vsC3	3	18
E3vsD3	0	0

## Data Availability

Data are available on request from the authors.

## Supplementary Materials

The following supporting information can be downloaded at https://www.mdpi.com/article/10.3390/insects16020199/s1: Table S1: Primers for qPCR; and Table S2: Selected 25 genes and their abbreviations in immune-related pathways enriched at different infection stages.
