# Supplementary material for: Research on the Expression of Immune-Related Genes at Different Stages in the Third-Instar Larvae of Spodoptera frugiperda Infected by Metarhizium rileyi
Source: Insects. 2025 Feb 12;16(2):199. doi: 10.3390/insects16020199 (PMC11856804; doi:10.3390/insects16020199)
Supplement: Supplementary file 1 [file insects-16-00199-s001.zip › insects-3421757-supplementary.pdf]

Supplementary Table S1. Primers for qPCR.

**Primer name**	**Primer sequence** (5' to 3')
IDH3γ F Primer	GCGATGGCTCTGAGATTACTG
IDH3γ R Primer	GGACGAGGTGCTGCTTTCT
PGK1 F Primer	ACCCCTGGATCTGTAATCTTGC
PGK1 R Primer	TGCCCTGAATGCCTTGACC
CYP302A1 F Primer	CGAGGTACGGGAGTGACAAG
CYP302A1 R Primer	GGCGATAGTTCAGCCAAGC
FAH F Primer	AGGCATCAGACTCGTTCGG
FAH R Primer	ATCAAAAGGTATGGCAGGGA
ALDH7A1 F Primer	AGGGTCTGGCGGAGAAGTAG
ALDH7A1 R Primer	AGCAGGCAGCCTGTATGGA
CAT F Primer	AGTGGCTCTGCTGATACTGTCC
CAT R Primer	TGGGTATGGATGAAACTAGGGA
HEXB1 F Primer	TCGTGGTACTTGGACTATTTGG
HEXB1 R Primer	ACACTGCCTTTGGACTGCTC
LAMB1 F Primer	TTTACGACTTTCCGACCTACAA
LAMB1 R Primer	ACCTTCTGCCCGACTAACG
HEXB2 F Primer	AGTCTACCCAGCGGCACAA
HEXB2 R Primer	CAACAGCACTAGCACGAGGC
GBA F Primer	CCTACGCCGACTACCATTACA
GBA R Primer	TCCAGCCGAGGTTGTTGA
CP F Primer	AACCAAACATTTCCACTTCTCG
CP R Primer	CCAAGCTAACGTCGGCATA
NFKB1 F Primer	TTCATTCGTACCCTGCTGTTAG
NFKB1 R Primer	GCTGCATCCTGTCTCCTTGC
ALDOA1 F Primer	TCGCAAGATGAGGTTACGACT
ALDOA1 R Primer	GCCTGGACTGATGGGATGT
CYCS F Primer	GCAGGAAACGCTGAAAACG
CYCS R Primer	TCTTGCGACCCCAGAAACC
ALDH1A1 F Primer	GTCCCCTATGCCGCTAATG
ALDH1A1 R Primer	CGCCTTCTTGCTTTCCCT
FAR F Primer	TAAATGTGATCGTCCACCTAGC
FAR R Primer	TTCAGTCAATCGTTCTTCCTCC
GSTA1 F Primer	GCATTCCTGTCCCGTACAAA
GSTA1 R Primer	CTTCACAAACGCACCCAAA
UGT5 F Primer	AGGAGAATCTACCGAACCAGC
UGT5 R Primer	GAGGGATAGGAGACCACCGT
MSDH F Primer	AATCTTCGGACCTGTCATCG
MSDH R Primer	CGTTACCGTAGGGGTTTGAG

ALDOA2 F Primer	CCAGGATTTGGAACGAAGG
ALDOA2 R Primer	GGACTCATCAGCAGCAAGGA
XDH F Primer	TTTGTAGAGCACCAGGCACA
XDH R Primer	ACAGAAAGCCCATCTCAATCC
ARDH1 F Primer	GAAGGTCCACTACTCAGGCATC
ARDH1 R Primer	TTACGGCAGAAATCGCACA
GST1 F Primer	TCCTGGAAGCATTCTTGTCG
GST1 R Primer	AAGCCTTAGCACCTTCTTCATT
CYP307A1 F Primer	AAGATTAGGGCAGAGTTGGACG
CYP307A1 R Primer	CGCTACATGAGGTACGATGGG
NPC2 F Primer	TGCGACAGTACATATTATGGGG
NPC2 R Primer	GGACAGTGGGAGTTAGTGAGGT
RPS24 F Primer	CACTGGCTTTGCTCTCATC
RPS24 R Primer	TCATCCTGTTCTTGCGTTC

Supplementary Table S2. Selected 25 genes and their abbreviations in immune-related pathways enriched at different infection stages.

Stage	Pathway	Gene	Abbreviations
0–24 h post-infection	Carbon metabolism/Biosynthesis of amino acids	isocitrate dehydrogenase [NAD] subunit gamma	*IDH3γ*
	Glycolysis/Gluconeogenesis	phosphoglycerate kinase	*PGK1*
	Insect hormone biosynthesis	cytochrome P450 302a1	*CYP302A1*
	Tyrosine metabolism	fumarylacetoacetase hydrolase	*FAH*
	Purine metabolism	allantoicase	*ALLC*
	Peroxisome	catalase	*CAT*
	Glycosaminoglycan degradation/Lysosome	beta-hexosaminidase subunit beta	*HEXB1*
	ECM–receptor interaction	laminin subunit beta-1	*LAMB1*
24–48 h post-infection	Glycosaminoglycan degradation	beta-hexosaminidase subunit beta	*HEXB2*
	Lysosome	lysosomal acid glucosylceramidase-like	*GBA*
	ECM–receptor interaction	laminin subunit beta-1	*LAMB1*

	Autophagy—animal	cathepsin B-like	*CP*
	Toll and Imd signaling pathways	NF-kappa-B p110 subunit	*NF-KB1*
	Carbon metabolism/Pentose phosphate pathway/Biosynthesis of amino acids	fructose-bisphosphate aldolase	*ALDOA*
	Oxidative phosphorylation	cytochrome c	*CYCS*
	Glycolysis/Gluconeogenesis/Ascorbate and aldarate metabolism	aldehyde dehydrogenase 1A1	*ALDH1A1*
	Peroxisome	fatty acyl-CoA reductase wat	*FAR*
	Drug metabolism—cytochrome P450/Metabolism of xenobiotics by cytochrome P450/Glutathione metabolism	glutathione S-transferase 1	*GSTA1*
	Pentose and glucuronate interconversions	UDP-glycosyltransferase UGT5-like	*UGT5*
	Tyrosine metabolism	fumarylacetoacetase hydrolase	*FAH*
48–72 h post-infection	Carbon metabolism	probable methylmalonate-semialdehyde dehydrogenase [acylating]	*MSDH*
	Glycolysis/Gluconeogenesis/Insect hormone biosynthesis/Ascorbate and aldarate metabolism	aldehyde dehydrogenase 1A1	*ALDH1A1*
	Pentose phosphate pathway/Biosynthesis of amino acids	fructose-bisphosphate aldolase	*FBA*
	Peroxisome	xanthine dehydrogenase 1-like	*XDH*
	Pentose and glucuronate interconversions	D-arabinitol dehydrogenase 1-like	*DADH*
	Glutathione metabolism/Drug metabolism—cytochrome P450/Metabolism of xenobiotics by cytochrome P450	glutathione S-transferase 1	*GST1*
	Tyrosine metabolism	fumarylacetoacetase hydrolase	*FAH*
72–96 h post-infection	Insect hormone biosynthesis	cytochrome P450 307a1	*CYP307A1*
	Lysosome	NPC intracellular	*NPC2*

		cholesterol transporter 2-like	
	Glycosaminoglycan degradation	beta-hexosaminidase subunit beta	*HEXB2*
